# sTREM2 mediates the associations of minimal depressive symptoms with amyloid pathology in prodromal Alzheimer’s disease: The CABLE study

**DOI:** 10.1038/s41398-022-01910-4

**Published:** 2022-04-04

**Authors:** Zhi-Bo Wang, Yan Sun, Ya-Hui Ma, Yan Fu, Hao Hu, Wei Xu, Zuo-Teng Wang, Ling-Zhi Ma, Lan Tan, Jin-Tai Yu

**Affiliations:** 1 Department of Neurology, Qingdao Municipal Hospital, Qingdao University, Qingdao, China; 2 Department of Neurology, Qingdao Municipal Hospital, College of Medicine and Pharmaceutics, Ocean University of China, Qingdao, China; 3 Department of Neurology and Institute of Neurology, Huashan Hospital, State Key Laboratory of Medical Neurobiology and MOE Frontiers Center for Brain Science, Shanghai Medical College, Fudan University, Shanghai, China

**Keywords:** Depression, Learning and memory

## Abstract

The effects of microglial activation on the associations between depression and Alzheimer’s disease (AD) are still unclear. *TREM2* gene plays a pivotal role in microglial activation, has been identified as a risk factor for AD. In this work, we aimed to assess the interrelationships of soluble TREM2 (sTREM2) level in cerebrospinal fluid (CSF), minimal depressive symptoms (MDSs), and CSF amyloid markers. The linear regression analyses were conducted on 796 cognitively unimpaired participants from the CABLE (Chinese Alzheimer’s Biomarker and LifestylE) study. Causal mediation analyses with 10,000 bootstrapped iterations were used to test the mediation effects. In addition, similar statistical analyses were performed in subgroups stratified by sex, age, and *APOE ε4* carrier status. In total subjects, MDSs were associated with lower CSF sTREM2 levels (*p* < 0.0001), lower CSF amyloid markers (*p* < 0.0001), and poorer cognitive performance (MMSE, *p* = 0.0014). The influence of MDSs on CSF amyloid markers was partially mediated by CSF sTREM2 (proportion from 2.91 to 32.58%, *p* < 0.0001). And we found that the sTREM2-amyloid pathway partially mediated the effects of MDSs on cognition. Of note, exploratory subgroup analyses showed that the above influences of CSF sTREM2 were pronounced in the *APOE ε4* (−) group. These results suggest that early depression is associated with amyloid pathology, which might be partly mediated by microglial activation, especially in the absence of *APOE ε4*.

## Introduction

Alzheimer disease (AD) is the most common cause of dementia, and nearly all patients had accompanied neuropsychiatric symptoms in the very early stage of the disease [1]. Depression as one of the most frequent neuropsychiatric symptoms is a common risk factor related to the incidence of AD [2, 3]. A longitudinal study reported that participants with depressive symptoms are at a high risk of dementia over a 20-year follow-up [4]. However, the biological mechanism underlying the association between depression and dementia is still unclear. Recent evidence suggests that depressive symptoms together with aberrant Aβ accumulation could result in cognitive impairment and the development of dementia [5]. And the minimal depressive symptoms (MDSs), a symptom prior to subclinical depressive symptoms, were associated with abnormal amyloid pathologies and cognitive impairment [6].

Previous studies have reported that activation of immune mediators is an important regulator of the AD pathology, and dysfunction of immune-microglia molecular network is one pathogenesis of AD [7] as well as of depression [8]. *TREM2* is an immune-related gene expressed in microglia, promoting microglial phagocytosis, chemotaxis, and survival [9, 10] and regulating the transition of microglia from homeostatic to disease-associated microglia (DAM) [11]. The soluble TREM2 (sTREM2), which is shed from DAM following cleavage of TREM2, has been identified as a novel cerebrospinal fluid (CSF) biomarker in AD [12, 13]. In prodromal AD, higher CSF sTREM2 was associated with larger gray matter volume [14], lower amyloid pathology [15], and greater cognitive function, and slower subsequent clinical progression [16]. Together, these findings indicate a protective role of increased sTREM2 in early stage of AD. In addition, a significant reduction in CSF sTREM2 was found in major depressive disorder (MDD) [17], suggesting that CSF sTREM2 which represented neuroinflammation might be involved in the pathogenesis of depression as well. However, whether CSF sTREM2 influences the association of depressive symptoms and cerebral amyloid pathology in the preclinical stage of AD is still unclear.

Therefore, we aimed: (1) to analyze the differences in CSF sTREM2 between participants with MDS and normal participants; (2) to explore the interrelationship of MDS with CSF sTREM2 and CSF amyloid markers, as well as its interaction with age, sex, and *APOE ε4* status; (3) to assess whether the association of MDS with amyloid pathology was mediated by CSF sTREM2. To achieve these aims, we used MDS to explore these associations since it represents an early manifestation of depression, and all these analyses were performed in non-demented participants since they were at the preclinical stage of AD, which allowed us to investigate very early inflammatory changes associated with early stages of depression.

## Materials and methods

### The CABLE database

All non-demented participants in this study were included from the Chinese Alzheimer’s Biomarker and Lifestyle (CABLE) cohort, which was an ongoing large and independent cohort focusing primarily on the biomarkers and risk factors of AD. Participants in the CABLE study were recruited from Qingdao Municipal Hospital, Shandong, China. All eligible participants were required to be 40–90 years old and to be of Han Chinese origin. Participants with a history of major neurological disorders, major psychological disorders, malignant tumors, or genetic disorders were excluded. In this study, additional exclusion criteria also included the medical history and medication records of depression. All enrolled participants were required to undergo comprehensive clinical, neuropsychological, psychosocial, and psychiatric evaluations, and their biological sample (blood and CSF sample) were collected by doctors with standardized training. The CABLE database was approved by the ethic committee of Qingdao Municipal Hospital and was accorded with the Helsinki Declaration. All the participants provided written informed consent.

### Measurements of cognition and neuropsychiatric symptoms

Depressive symptoms were measured using the 17-item version of the Hamilton Depression Rating Scale. Eligible participants were classified into normal group (absence of depressive symptoms) and MDS group. Our definition of MDSs in participants was consistent with its definition as a Hamilton Depression Rating Scale score ≥1 and ≤7 by previous studies [18, 19]. The Chinese version of Mini-Mental State Examination (MMSE) was used to assess the global cognitive function of participants in CABLE.

### Measurements of CSF AD biomarkers and CSF sTREM2

CSF samples were collected by lumbar puncture into 10 ml polypropylene tubes, and they were sent to the lab within 2 h. All CSF samples were centrifuged at 2000 × *g* for 10 min and stored in freezers at −80 ˚C. Before testing CSF samples, the thaw/freezing cycle was controlled within 2 cycles. CSF AD biomarkers (Aβ_1-42_, Aβ_1-40_, tau, and phosphorylated tau_181_ [p-tau_181_]) and CSF sTREM2 were measured on the microplate reader (Multiskan MK3; Thermo Fisher Scientific, Waltham, MA). CSF sTREM2 measurements were conducted using the enzyme-linked immunosorbent assay (ELISA) kit (Human TREM2 SimpleStep ELISA kit; Abcam, no. Ab224881), and CSF AD biomarker measurements were done with other ELISA kits (Innotest; Fujirebio, Ghent, Belgium). All ELISA measurements were tested by experienced technicians who were blinded to the basic information of patients. CSF samples and standards were measured in duplicate, and the means of duplicates were used for the statistical analyses. The within-batch precision values were <5% for all proteins and the inter-batch coefficients of variation were <15%. The ratio of Aβ42 to Aβ40 was used to assess the levels of pathologic species while accounting for individual differences in amyloid production. The t-tau/Aβ_42_ and p-tau/Aβ_42_ ratios were used to predict cerebral Aβ deposition and cognitive decline because they were considered as better predictors than those expressed alone [20–22].

### *APOE ε4* genotyping

The blood samples were used to extract DNA using QIAamp DNA Blood Mini Kit (Qiagen, Hilden, Germany). The extracted DNA was amplified by the polymerase chain reaction with forward primers 5′-ACGGCTGTCCAAGGAGCTG-3′ (rs429358) and 5′-CTCCGCGATGCCGATGAC-3′ (rs7412). The extracted DNA was then separated and stored at −80 °C until the *APOE ε4* genotyping was conducted with restriction fragment length polymorphism technology.

### Statistical analyses

Extreme values of CSF biomarkers were excluded (outside three SDs). We evaluated the normality of distribution for continuous variables using Shapiro–Wilk test, and those that did not follow a normal distribution were normalized by the Box-Cox transformations via “car” package of R software and were standardized by z-scale. The differences in baseline characteristics were tested by $\chi^{2}$ test (for categorical variables) and Mann–Whitney *U* test (for continuous variables).

Data Analyses with Bootstrap-coupled ESTimation (DABEST) were used to compare the differences in CSF sTREM2 levels [23]. These DABEST plots can visualize the effect size by plotting the data as the mean difference in CSF sTREM2 between normal group and MDS group. Multiple linear regressions (MLR) were used to explore the relationships of MDSs (independent variable) with CSF sTREM2, CSF AD boimarkers (Aβ_1-42_, tau, and p-tau_181_), and cognition (dependent variables) in total participants. Sex, age, education, and *APOE ε4* status were added as covariates in all MLR models. In addition, we further extended our analyses into subgroups stratified by sex, age (<65 years or ≥65 years), and *APOE ε4* status (have no or one/two *APOE ε4* allele) to analyze the differences between normal group and MDS group.

Mediation analyses of the single mediator (“mediate” package in R 4.1.0 software) were used to assess whether CSF sTREM2 could modulate the association between MDSs and amyloid pathology based on the method proposed by Baron and Kenny [24]. For each mediation model using CSF sTREM2 as the mediator, the following requirements must be reached: (1) MDSs were significantly associated with CSF sTREM2; (2) MDSs were significantly associated with CSF amyloid markers; (3) CSF sTREM2 was significantly associated with CSF amyloid markers, and (4) the associations between MDSs and CSF amyloid markers were attenuated when CSF sTREM2 was added in the regression model. Furthermore, CSF sTREM2 and CSF amyloid biomarkers were included as mediators to explore the effects of MDSs on cognition. In each model, MDS was included as an independent variable, and CSF amyloid biomarkers and cognitive scores were included as dependent variables. We used the PROCESS version 3.5.3 toolbox from SPSS (www.processmacro.org) to perform these multiple mediator models [25]. With the significance determined by 10,000 bootstrapped resamples, we estimated the attenuation or indirect effect. Each path of the mediation models was adjusted for the same covariates as previous MLR models.

A two-sided *p* value < 0.05 was considered statistically significant. The “dabestr”, “lm”, “mediate”, and “car” packages in R version 4.1.0 software and IBM SPSS Statistics 23 were used to perform the above analyses. The R version 4.1.0 software and GraphPad Prism version 9.00 were used for the above figure preparation.

## Results

### Characteristics of participants

A total of 796 participants were included in this study, of whom 140 had MDSs. The total participants had a mean age of 61.6 years old, a female proportion of 44%, and an *APOE ε4* carrier proportion of 17.5%. Individuals with MDSs tended to be older and they were more likely to have lower levels of CSF sTREM2, poorer cognitive performance, and greater amyloid deposition (Table 1). The detailed information about the participants in subgroups was presented in Table S1. No intragroup differences in CSF sTREM2 were found in the subgroups (Fig. S2), implying that there was no selection bias in the present study.

**Table 1:** Characteristics of participants.

	Total	Normal	MDSs^a^	*p* value^b^
*N*	796	656	140	-
Age, year, mean ± SD	61.6 ± 10.2	61.3 ± 10.1	63 ± 10.3	**0.037**
Sex, *n*_Male_/*n*_Female_	446/350	374/282	71/69	0.227
Education, years, mean ± SD	9.5 ± 4.3	9.5 ± 4.3	9.4 ± 4.4	0.911
Positive *APOEε4* carrier status, *n*(%)	139 (17.5)	119 (18.1)	20 (14.3)	0.275
MMSE scores, mean ± SD	27.2 ± 3.1	27.3 ± 3	26.6 ± 3.2	**0.003**
Depressive symptoms	0 (0–0)	0 (0–0)	2 (1–3)	**<0.001**
sTREM2 Mean ± SD	17605 ± 7206.6	18172.9 ± 7068.5	14945.1 ± 7276.3	**<0.001**
*CSF AD biomarkers and rations, mean ± SD*				
Aβ42 pg/mL	172.4 ± 90.8	181.4 ± 94.6	129.9 ± 52.7	**<0.001**
Aβ40 pg/mL	6057.1 ± 2599.2	5969.8 ± 2557.8	6466.3 ± 2757.7	**0.023**
Tau pg/mL	169.6 ± 72.5	170 ± 74.2	167.7 ± 63.9	0.729
P-tau pg/mL	37.3 ± 9	37.2 ± 9.2	37.6 ± 8.2	0.222
Aβ42/Aβ40 ratio	0.034 ± 0.026	0.035 ± 0.027	0.025 ± 0.018	**<0.001**
Tau/Aβ42 ratio	1.142 ± 0.616	1.089 ± 0.607	1.389 ± 0.601	**<0.001**
P-tau/Aβ42 ratio	0.254 ± 0.101	0.241 ± 0.098	0.313 ± 0.093	**<0.001**

Bold indicated that differences in normal group and MDS group were significant.

Abbreviations: *MDSs* minimal depressive symptoms, *APOE* apolipoprotein gene, *MMSE* Mini-Mental State examination, *sTREM2* soluble triggering receptor expressed on myeloid cells 2, *CSF* cerebrospinal fluid, *Aβ* amyloid-β, *P-tau* phosphorylated tau.

^a^MDSs were defined as 1≤ HAMD score ≤7.

^b^Significant differences in normal group and MDS group were used by the Mann–Whitney U test (for continuous variables) and Pearson’s $\chi^{2}$ test (for categorical variables).

### MDSs were associated with lower levels of CSF sTREM2 independent of anxiety symptoms and cognitive function

**Fig. 1:**
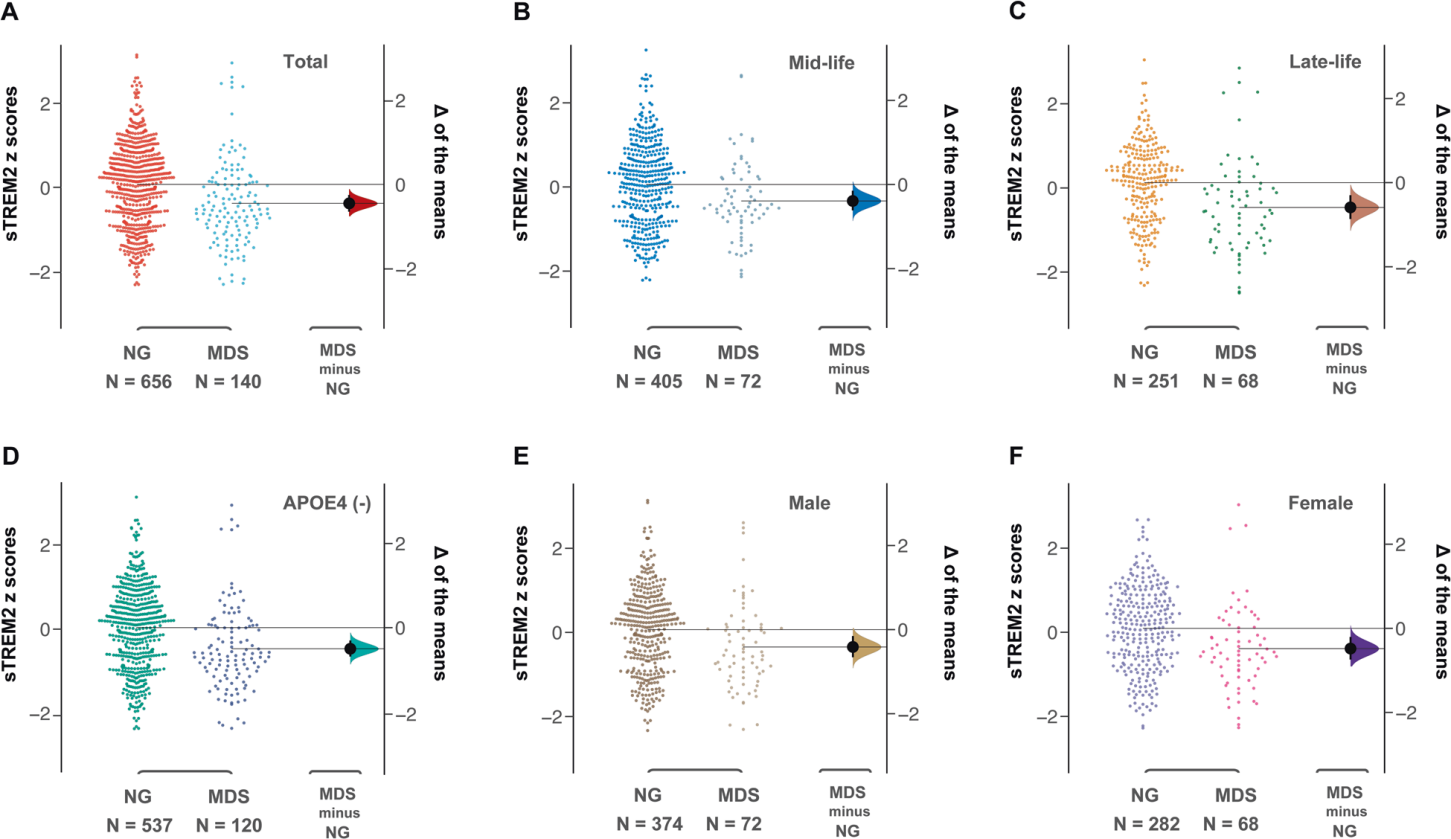
Differences of CSF sTREM2 in MDSs and normal participants. Levels of CSF sTREM2 were significant lower in MDSs participants than normal participants in total (**A**), mid-life (**B**), late-life (**C**), *APOE ε4* non-carriers (**D**), male (**E**), and female (**F**). The left panel shows the distribution of raw data points for the entire dataset and the right panel shows the differences by using 5000 bootstrapped resamples and with difference-axis origin aligned to the mean of the normal group distribution. For each estimation plot: black dot represents mean difference (as indicated in right panel), black ticks indicate 95% confidence interval, and the shaded area represents bootstrapped sampling error distribution. CSF cerebrospinal fluid; sTREM2 soluble of trigging receptor expressed on myeloid cells 2; MDS minimal depressive symptom; APOE apolipoprotein E.

We found that participants with MDSs had a significantly lower CSF sTREM2 than those in the normal group (95% CI = [−0.628; −0.251]) (Fig. 1A). To investigate whether a decreased level of CSF sTREM2 in MDS group can be observed in these subgroups, we extended the analysis into subgroups (Fig. 1B–F and Fig. S1). Lower levels of CSF sTREM2 in MDS participants were found in mid-life (95% CI = [−0.615; −0.149]), late-life (95% CI = [−0.865; −0.299]), male (95% CI = [−0.655; −0.152]), female (95% CI = [−0.741; −0.205]), and *APOE ε4* non-carrier (95% CI = [−0.688; −0.305]) subgroups, but not in the *APOE ε4* carrier subgroup (95% CI = [−0.642; 0.364]).

**Fig. 2:**
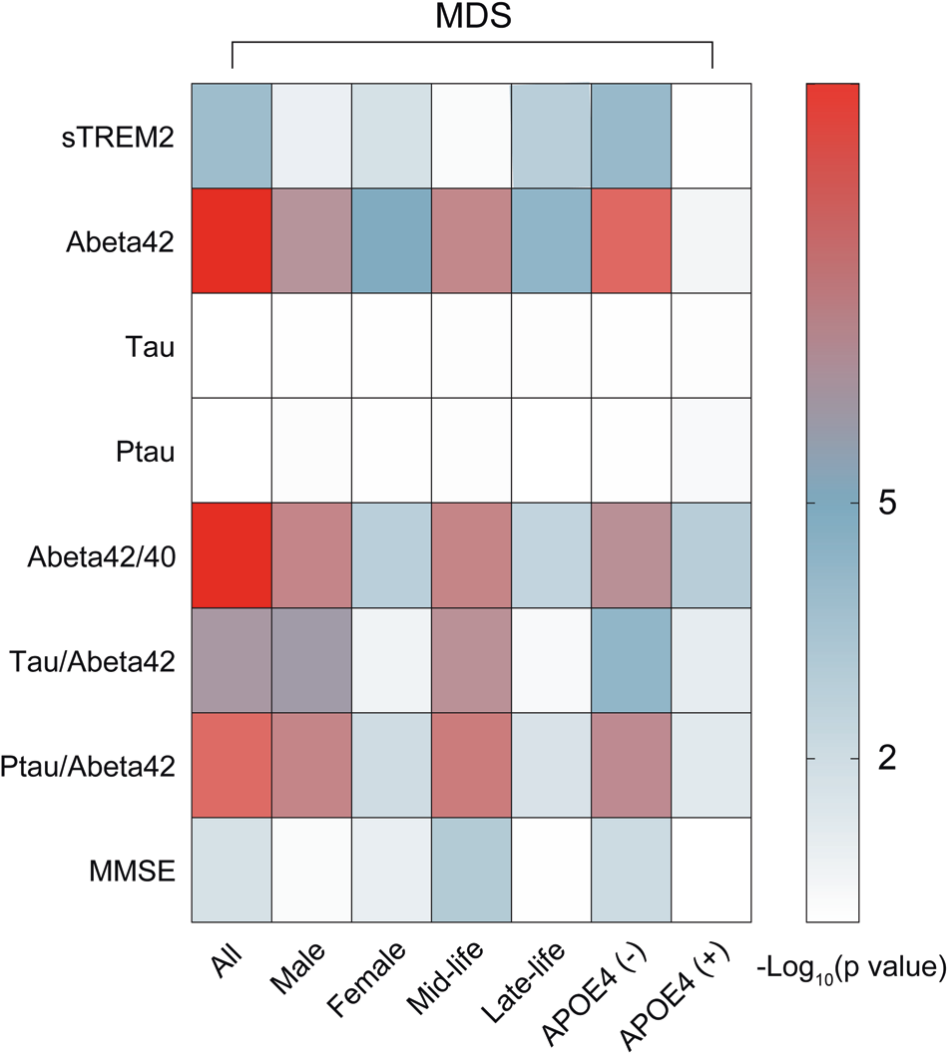
Association of MDSs with CSF biomarkers and cognition. MDSs were significant associated with CSF sTREM2, CSF amyloid markers, and cognition in total and subgroups, but not with CSF tau and CSF p-tau. MDSs minimal depressive symptoms; APOE apolipoprotein gene; MMSE Mini-Mental State examination; CSF cerebrospinal fluid; sTREM2 soluble triggering receptor expressed on myeloid cells 2; Aβ amyloid-β; P-tau phosphorylated tau.

We further explored the association between MDSs and CSF sTREM2 by using MLR models (Fig. 2). In total participants, we found that MDSs were significantly associated with lower CSF sTREM2 (*p* = 4.34 × 10^−5^), and a similar association existed in male (*p* = 0.0097), female (*p* = 0.0012), late-life (*p* = 0.0001), and *APOE ε4* non-carrier (*p* = 3.16 × 10^−5^) subgroups, but not in mid-life and *APOE ε4* carrier subgroups (*p* > 0.05). Considering a significant association between depressive symptoms and anxiety symptoms (*p* < 2 × 10^−16^), we further added anxiety symptoms as a covariate in our model. Consistent with previous findings, we found the association between MDSs and CSF sTREM2 remained significant after controlling for anxiety symptoms or/and cognitive scores (Table S2). Furthermore, interaction analyses showed that the association was influenced by *APOE ε4* status (Table S3).

### MDSs were associated with abnormal amyloid pathology and cognitive decline but not with tau-related pathologies

We used MLR models to explore the associations of MDSs with CSF AD biomarkers and cognition. In these models, MDSs were found associated with Aβ42 (*β* = −0.199, *p* = 2.17 × 10^−11^; male), Aβ42/40 (*β* = −0.191, *p* = 6.79 × 10^−11^), Tau/Aβ42 (*β* = 0.147, *p* = 3.78 × 10^−7^), and Ptau/Aβ42 (*β* = 0.185, *p* = 2.9 × 10^−10^). In subsample analyses, the above associations remained significant (Table S4). As for cognition, an association between MDSs and lower MMSE scores was found in total participants (*β* = −0.081, *p* = 0.0014), which also existed in female (*β* = −0.087, *p* = 0.0009), mid-life (*β* = −0.144, *p* = 0.0002), and *APOE ε4* non-carrier subgroups (*β* = −0.1, *p* = 0.0006), rather than male, late-life, and *APOE ε4* carrier subgroups (all *p* > 0.05) (Table S4). However, we did not find significant associations between MDSs and CSF tau-related biomarkers (*p* > 0.05). Overall, these findings demonstrated that MDSs were significantly related with cerebral amyloid deposition and cognitive impairment in the early stage of AD.

### CSF sTREM2 mediates the association of MDSs with amyloid pathology

**Fig. 3:**
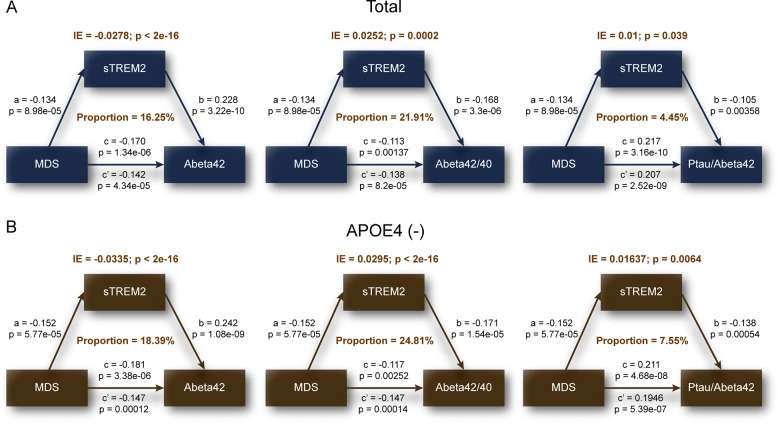
CSF sTREM2 mediated association between MDS and amyloid pathology. Models of mediation for MDS, CSF sTREM2, and CSF amyloid markers (ie. Aβ42, Aβ42/40, Ptau/Aβ42), with MDS as independent variable and CSF sTREM2 as mediator and CSF amyloid markers as dependent variable. Mediation effects of CSF sTREM2 are shown in total (**A**) and *APOE ε4* non-carriers (**B**). CSF cerebrospinal fluid; sTREM2 soluble of trigging receptor expressed on myeloid cells 2; MDS minimal depressive symptom; APOE apolipoprotein E. Aβ amyloid-β; P-tau phosphorylated tau; IE indirect effect.

Based on the preceding results, we then tested the main hypothesis around whether CSF sTREM2 was involved in the association between MDSs and amyloid pathology. We performed mediation analyses on CSF Aβ42, and the ratios of Aβ42/40 and Ptau/Aβ42 rather than the Tau/Aβ42 ratio because it did not show a significant association with CSF sTREM2 (Table S5). Results of mediation analyses showed that the association of MDSs and amyloid pathology was partially mediated by CSF sTREM2 in total participants (Fig. 3A), as well as in *APOE ε4* non-carrier (Fig. 3B), late-life (Fig. S3A), male (Fig. S3B), and female (Fig. S3C) subgroups, with the proportion of mediation varying from 2.91 to 32.58%. In addition, we found a three-step indirect effect of CSF sTREM2 and CSF amyloid markers in total participants (Fig. S4A) and *APOE ε4* non-carrier subgroup (Fig. S4B) (ie, MDSs → CSF sTREM2 → Aβ42/40 → MMSE).

## Discussion

**Fig. 4:**
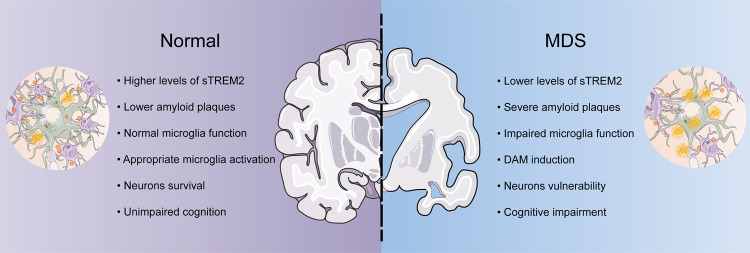
The role of CSF sTREM2 in MDS. The schematic graph is depicting the characteristic of association of MDS with CSF sTREM2 based on this study and current research in this filed. CSF cerebrospinal fluid; DAM disease-associated microglia; sTREM2 soluble of trigging receptor expressed on myeloid cells 2; MDS minimal depressive symptom.

This is the largest-scale study to assess the interrelationships between MDSs, CSF sTREM2, CSF amyloid biomarkers, and cognition among non-demented adults. Our primary result showed that the MDS group had significantly lower levels of CSF sTREM2 than the normal group. There was a strong association between MDSs and lower levels of CSF sTREM2, and the interaction analyses showed that *APOE ε4* status may modulate this association. We also found that the association of MDSs with amyloid pathology was partially mediated by CSF sTREM2. Of note, this mediation effect further led to cognitive decline. These findings support the hypothesis that lower CSF sTREM2 related to microglial activation is associated with abnormal amyloid pathology and worsened cognition in MDS participants (Fig. 4).

Our findings are consistent with a recent longitudinal study which showed that the levels of CSF sTREM2 were significantly lower in MDD individuals than controls [17], suggesting that microglial activation is accompanied with the pathogenesis of depression [8]. Multiple lines of evidence also supported the hypothesis that microglial activation is a central regulator of depression [26, 27]. Consistent with previous studies reporting the protective role of sTREM2 in AD [28–30], our findings indicated that lower levels of CSF sTREM2 in the MDS group exerted modulatory effects on cerebral amyloid accumulation, which reinforced the regulatory role of neuroinflammation in the relationship between depression and AD [31]. Importantly, we previously reported that the relationship between depression and cognitive impairment was only mediated by amyloid pathology [6]. The results of our present study indicated that CSF sTREM2 was also a key mediator in this association. Thus, microglial activation might increase the risk of cognitive decline by promoting cerebral amyloid deposition among MDS subjects in the preclinical stage of AD.

Emerging evidence has demonstrated that epidemiological sex differences in depression and dementia are well characterized, and to be specific, both diseases are more common in females than males [32]. Previous studies reported that females had higher inflammatory responses which were related with severe depressive symptoms [33–35]. While we found no sex differences in the association of MDS and CSF sTREM2, our findings showed that MDSs had stronger associations with impaired microglial function and increased amyloid burdens in females than males based on the proportion of mediation effects (maximum 24.27% vs maximum 17.93%), which might explain females’ greater susceptibility to AD.

Several studies suggested that the incidence of depression has reached its peak in people aged 80 years or older [36], and late-life depression (LLD) could serve as a prodrome of AD [37]. In our present study, we found that the association of depressive symptoms with CSF sTREM2 were only significant in older participants (≥65 years). This is in line with a recent study which showed that changes in plasma immunological protein were related with age and depression [38], suggesting that depressive symptoms in older people might have stronger inflammatory responses which increased their physical and psychological vulnerability to neurodegeneration.

*APOE ε4* allele is thought to be implicated in microglial responses and it has been identified as a risk factor for depression and AD [7, 39]. In the present study, we found an interaction effect between depressive symptoms and *APOE ε4* on CSF sTREM2, and the association between MDSs and CSF sTREM2 was only significant in *APOE ε4* non-carriers. To our knowledge, this has not been reported before. Multiple hypotheses may explain these observations. There is an over-activated microglial response that may be presented prior to the onset of depressive symptoms due to the role of *APOE ε4* in systemic inflammatory response [7]. At this stage of pathogenesis, significant inflammatory responses have occurred and would likely be very difficult to be influenced by MDS. Besides, it is possible that the higher affinity of sTREM2-*APOE ε4* complexes may disturb depression-sTREM2 pathway in the presence of *APOE ε4* [40, 41], as indicated by C1q-APOE complexes [42]. However, the specific biological mechanism in this association still needs to be explored in future study.

Several biological mechanisms have been proposed to explain how depressive symptoms affect CSF sTREM2 and amyloid pathology. Firstly, persistent chronic stresses with depressive-like condition have been implicated in impaired microglial function and suppressed hippocampal neurogenesis [43], and then accelerated aggregation of toxic proteins, like Aβ and tau protein. Secondly, numerous inflammatory mediators thought to be implicated in immune-microglial molecular network have been identified as risk factors in depression [8], and sTREM2 may be a downstream factor to those inflammatory mediators. Finally, a reversed result suggests that depressive symptoms may be the results of amyloid pathology in the progression of AD [44], suggesting that aggregation of amyloid burdens is possible to damage microglia homeostatic functions and to enhance related inflammatory responses, and therefore, may be increased the incident risk of depression and dementia.

There are two strengths of this study. First, the use of non-demented participants made our results highly sensitive to the preclinical stage of AD. Second, we were the first to reveal the impact of sTREM2 on the relationship between depression and amyloid pathology. However, some limitations also need to be taken into account in this study. Firstly, depression in this study was restricted to MDSs, which may limit the interpretability of associated results with depression. Secondly, the question that whether CSF sTREM2 represents a direct measure of TREM2 or a soluble form of TREM2 is still controversial. Thirdly, all our findings are based on CSF data that are less accurate than PET imaging for measuring cerebral neurodegeneration. Last, although our models suggested a mediation analysis best described the association between depression and amyloid pathology, we cannot conclude the causality of this association due to the limitation of mediation analysis and cross-sectional data that we use.

In conclusion, our results showed that CSF sTREM2 is a key mediator in the associations of depression with amyloid pathology and cognitive function. This provided clinical implications for the treatment of depressive individuals in the prodromal stage of AD, since targeting sTREM2 may reduce amyloid deposition and improve cognitive function. Together, our results indicated that TREM2 could serve as a promising target for future AD treatment.

## Supplementary information

Supplementary materials
