# Supplementary material for: sTREM2 mediates the associations of minimal depressive symptoms with amyloid pathology in prodromal Alzheimer’s disease: The CABLE study
Source: Transl Psychiatry. 2022 Apr 4;12:140. doi: 10.1038/s41398-022-01910-4 (PMC8980028; doi:10.1038/s41398-022-01910-4)
Supplement: Supplementary file 1 — Supplementary materials [file 41398_2022_1910_MOESM1_ESM.docx]

**sTREM2 mediates the associations of minimal depressive symptoms with amyloid pathology in prodromal Alzheimer’s disease: The CABLE study**

Zhi-Bo Wang, MD^1,#^, Yan Sun, MD, PhD^1,#^, Ya-Hui Ma, MD, PhD^1^, Yan Fu, MD^1^, Hao Hu, MD, PhD^1^, Wei Xu, MD, PhD^1^, Zuo-Teng Wang, MD, PhD^2^, Ling-Zhi Ma, MD, PhD^1^, Prof. Lan Tan^1,2,*^, Prof. Jin-Tai Yu^3,*^

^1^ Department of Neurology, Qingdao Municipal Hospital, Qingdao University, Qingdao, China.

^2^ Department of Neurology, Qingdao Municipal Hospital, College of Medicine and Pharmaceutics, Ocean University of China, Qingdao, China.

^3^ Department of Neurology and Institute of Neurology, Huashan Hospital, State Key Laboratory of Medical Neurobiology and MOE Frontiers Center for Brain Science, Shanghai Medical College, Fudan University, Shanghai, China.

**Table legends**

|  | **Male** | | **Female** | | **Midlife** | | **Late-life** | | ***APOE ε4* (+)** | | ***APOE ε4* (-)** | |
| --- | --- | --- | --- | --- | --- | --- | --- | --- | --- | --- | --- | --- |
|  | **Normal** | **MDSs** | **Normal** | **MDSs** | **Normal** | **MDSs** | **Normal** | **MDSs** | **Normal** | **MDSs** | **Normal** | **MDSs** |
| N | 374 | 72 | 282 | 68 | 405 | 72 | 251 | 68 | 119 | 20 | 537 | 120 |
| Age, Year, Mean ± SD | 61.5 ± 10.7 | 65.2 ± 10.3 | 60.9 ± 9.3 | 60.8 ± 9.9 | 55.0 ± 6.9 | 55 ± 7.1 | 71.3 ± 5.3 | 71.6 ± 4.9 | 60.4 ± 10.6 | 65.5 ± 9.1 | 61.5 ± 10.0 | 62.6 ± 10.5 |
| Sex, *n*_Male_/*n*_Female_ | 374/0 | 72/0 | 0/282 | 0/68 | 170/235 | 44/28 | 112/139 | 24/44 | 43/76 | 8/12 | 239/298 | 60/60 |
| Education, Years, Mean ± SD | 10.2 ± 4.0 | 10.4 ± 3.7 | 8.7 ± 4.5 | 8.3 ± 4.8 | 10.4 ± 4.1 | 9.9 ± 4.6 | 8.2 ± 4.4 | 8.8 ± 4.2 | 9.9 ± 3.9 | 9.0 ± 4.5 | 9.5 ± 4.4 | 9.4 ± 4.4 |
| *APOEε4* Carrier *n*(％) | 76 (20.3) | 12 (16.7) | 43 (15.2) | 8 (11.8) | 79 (19.5) | 9 (12.5) | 40 (15.9) | 11 (16.2) | 119 (100) | 20 (100) | 0 (0) | 0 (0) |
| MMSE Scores, Mean ± SD | 27.5 ± 2.9 | 27 ± 2.8 | 27.1 ± 3.2 | 26.2 ± 3.6 | 27.8 ± 2.8 | 26.8 ± 2.8 | 26.6 ± 3.3 | 26.4 ± 3.7 | 26.6 ± 2.4 | 26.7 ± 3.9 | 27.3 ± 3.1 | 26.6 ± 3.1 |
| Depressive Symptoms | 0 (0-0) | 2 (1-3) | 0 (0-0) | 2 (1-4) | 0 (0-0) | 2 (1-3) | 0 (0-0) | 2 (1-3) | 0 (0-0) | 3 (2-5) | 0 (0-0) | 2 (1-3) |
| sTREM2 Mean ± SD | 18313.2 ± 7096.8 | 15340.4 ± 7388.1 | 17986.7 ± 7039.2 | 14526.6 ± 7186.8 | 17229.0 ± 7171.9 | 14418.6 ± 6631.4 | 19695.8 ± 6634.3 | 15502.6 ± 7913.6 | 18042.6 ± 7231.6 | 17067.9 ± 8106.7 | 18201.2 ± 7038.4 | 14591.3 ± 7103.9 |
| CSF AD Biomarkers and Rations, Mean ± SD | | | | | | | | | | | | |
| Aβ42 | 186.4 ± 96.3 | 133.6 ± 60.6 | 174.9 ± 92.1 | 126.1 ± 42.7 | 181.8 ± 98.7 | 128.1 ± 57.0 | 180.8 ± 87.8 | 131.9 ± 48.0 | 161.4 ± 74.2 | 127.1 ± 45.8 | 185.8 ± 98.1 | 130.4 ± 53.9 |
| Aβ40 | 5836.6 ± 2472.0 | 6421.9 ± 2945.8 | 6146.4 ± 2661.6 | 6513.4 ± 2564.6 | 5719.0 ± 2562.6 | 6208.0 ± 2686.8 | 6374.4 ± 2502.5 | 6739.9 ± 2824.8 | 5534.2 ± 2302.6 | 7560.0 ± 2967.5 | 6066.3 ± 2603.1 | 6284.0 ± 2691.2 |
| tau | 166.6 ± 68.4 | 175.6 ± 72.6 | 174.6 ± 82.1 | 159.3 ± 52.3 | 152.8 ± 58.0 | 155.1 ± 54.4 | 197.8 ± 87.9 | 181.0 ± 70.5 | 165.0 ± 75.7 | 200.6 ± 74.2 | 171.1 ± 73.9 | 162.2 ± 60.6 |
| P-tau | 36.9 ± 8.8 | 38.3 ± 8.1 | 37.7 ± 9.7 | 37.0 ± 8.3 | 35.8 ± 8.3 | 36.2 ± 7.3 | 39.7 ± 10.1 | 39.1 ± 8.9 | 36.5 ±9.5 | 43.9 ± 10.4 | 37.4 ± 9.1 | 36.6 ± 7.3 |
| Aβ42/Aβ40 ratio | 0.038 ± 0.032 | 0.026 ± 0.018 | 0.032 ± 0.018 | 0.024 ± 0.018 | 0.037 ± 0.031 | 0.025 ± 0.162 | 0.032 ± 0.019 | 0.025 ± 0.020 | 0.037 ± 0.044 | 0.022 ± 0.020 | 0.035 ± 0.022 | 0.026 ± 0.018 |
| Tau/Aβ42 ratio | 1.046 ± 0.585 | 1.419 ± 0.629 | 1.145 ± 0.632 | 1.358 ± 0.572 | 0.983 ± 0.499 | 1.300 ± 0.504 | 1.259 ± 0.718 | 1.484 ± 0.68 | 1.179 ± 0.713 | 1.649 ± 0.671 | 1.069 ± 0.580 | 1.346 ± 0.580 |
| P-tau/Aβ42 ratio | 0.234 ± 0.100 | 0.313 ± 0.085 | 0.250 ± 0.096 | 0.314 ± 0.101 | 0.232 ± 0.087 | 0.307 ± 0.085 | 0.256 ± 0.113 | 0.320 ± 0.100 | 0.260 ± 0.111 | 0.368 ± 0.117 | 0.237 ± 0.095 | 0.304 ± 0.085 |

**Table S1: Characteristics of subgroups**

Abbreviations: MDSs, minimal depressive symptoms; APOE, apolipoprotein E; MMSE, Mini-Mental State examination; CSF, cerebrospinal fluid; sTREM2, soluble triggering receptor expressed on myeloid cells 2; Aβ, amyloid-β; P-tau, phosphorylated tau.

|  | Model 1 | |  | Model 2 | |  | Model 3 | |
| --- | --- | --- | --- | --- | --- | --- | --- | --- |
|  | *β* | *P* |  | *β* | *P* |  | *β* | *P* |
| Total^#^ | -0.1192 | **4.5E-05** |  | -0.1066 | **2.9E-03** |  | -0.1113 | **1.9E-03** |
| Male^*^ | -0.1140 | **9.03E-03** |  | -0.1173 | **0.0259** |  | -0.1214 | **0.0211** |
| Female^*^ | -0.1227 | **1.73E-03** |  | -0.0994 | **0.0421** |  | -0.1027 | **0.0364** |
| Mid-life**^†^** | -0.0744 | 0.0872 |  | -0.0636 | 0.2601 |  | -0.0843 | 0.1378 |
| Late-life**^†^** | -0.1585 | **1.37E-04** |  | -0.1481 | **2.77E-03** |  | -0.1488 | **2.59E-03** |
| *APOE ε4* non-carriers**^‡^** | -0.1434 | **2.11E-05** |  | -0.1207 | **2.15E-03** |  | -0.1302 | **9.75E-04** |
| *APOE ε4* carriers**^‡^** | -0.0503 | 0.3861 |  | -0.0592 | 0.511 |  | -0.0688 | 0.452 |

**Table S2: Association of minimal depressive and anxiety symptoms with CSF sTREM2**

Independent variable was MDS and dependent variable was CSF sTREM2. Effect sizes are calculated as standardized betas. Significant effects (p<0.05) are shown in bold.

#Adjusting for age, sex, APOE ε4 status, and education.

*Adjusting for age, APOE ε4 status, and education.

† Adjusting for sex, APOE ε4 status, and education.

‡ Adjusting for age, sex, and education.

Model 1: baseline covariables + further adjusting MMSE scores.

Model 2: baseline covariables + further adjusting anxiety symptoms.

Model 3: baseline covariables + further adjusting MMSE scores and anxiety symptoms.

Abbreviations: CSF, cerebrospinal fluid; sTREM2, soluble of trigging receptor expressed on myeloid cells 2; APOE, apolipoprotein E; MDS, minimal depressive symptom.

|  | CSF sTREM2 | |
| --- | --- | --- |
|  | *β* | *P* |
| MDS | -0.114 | **8.98E-05** |
| MDS×Age | -0.005 | 0.113 |
| MDS×Sex | 0.003 | 0.963 |
| MDS×Education | -0.012 | 0.095 |
| MDS×*APOE ε4* | 0.116 | **0.0359** |

**Table S3: Interactions analyses results**

Adjusting for age, sex, APOE ε4 status, and education.

Abbreviations: CSF, cerebrospinal fluid; sTREM2, soluble of trigging receptor expressed on myeloid cells 2; MDS, minimal depressive symptom; APOE, apolipoprotein E.

|  | Aβ42 | |  | Aβ42/Aβ40 | |  | Tau/Aβ42 | |  | pTau/Aβ42 | |  | MMSE | |
| --- | --- | --- | --- | --- | --- | --- | --- | --- | --- | --- | --- | --- | --- | --- |
|  | *β* | *P* |  | *β* | *P* |  | *β* | *P* |  | *β* | *P* |  | *β* | *P* |
| Male^*^ | -0.232 | **1.4E-07** |  | -0.242 | **2.08E-08** |  | 0.211 | **7.97E-07** |  | 0.243 | **1.86E-08** |  | -0.067 | 0.0838 |
| Female^*^ | -0.178 | **1.08E-05** |  | -0.151 | **0.0002** |  | 0.094 | **0.0185** |  | 0.137 | **0.0007** |  | -0.087 | **0.009** |
| Mid-life**^†^** | -0.22 | **2.54E-07** |  | -0.238 | **2.18E-08** |  | 0.229 | **9.11E-08** |  | 0.249 | **5.52E-09** |  | -0.144 | **0.0002** |
| Late-life**^†^** | -0.173 | **2.68E-05** |  | -0.147 | **0.0004** |  | 0.079 | 0.0565 |  | 0.127 | **0.002** |  | -0.034 | 0.368 |
| *APOE ε4* non-carriers**^‡^** | -0.216 | **2.45E-10** |  | -0.18 | **1.12E-07** |  | 0.142 | **2.53E-05** |  | 0.187 | **5.10E-08** |  | -0.1 | **0.0006** |
| *APOE ε4* carriers**^‡^** | -0.137 | **0.0238** |  | -0.214 | **0.0002** |  | 0.152 | **0.0063** |  | 0.163 | **0.0038** |  | -0.017 | 0.7425 |

**Table S4: Association of minimal depressive symptoms with amyloid markers and cognitive function**

Independent variable was MDS. Dependent variables were CSF amyloid markers and were normalized by log-transformed and standardized by z-scale. Significant effects (p<0.05) are shown in bold.

*Adjusting for age, APOE ε4 status, and education.

†Adjusting for sex, APOE ε4 status, and education.

‡Adjusting for age, sex, and education.

Abbreviations: Aβ, amyloid-β; P-tau, phosphorylated tau; CSF, cerebrospinal fluid; APOE, apolipoprotein E; MDS, minimal depressive symptom; MMSE, Mini-Mental State examination.

|  | Aβ42 | |  | Aβ42/Aβ40 | |  | Tau/Aβ42 | |  | pTau/Aβ42 | |  | MMSE | |
| --- | --- | --- | --- | --- | --- | --- | --- | --- | --- | --- | --- | --- | --- | --- |
|  | *β* | *P* |  | *β* | *P* |  | *β* | *P* |  | *β* | *P* |  | *β* | *P* |
| Total^#^ | 0.271 | **5.26E-14** |  | -0.127 | **0.0004** |  | 0.02 | 0.5786 |  | -0.109 | **0.0025** |  | -0.029 | 0.344 |
| Male^*^ | 0.247 | **2.5E-07** |  | -0.159 | **0.0008** |  | 0.033 | 0.489 |  | -0.103 | **0.03** |  | -0.046 | 0.275 |
| Female^*^ | 0.298 | **6.43E-08** |  | -0.09 | 0.1092 |  | 0.005 | 0.9329 |  | -0.111 | **0.0474** |  | -0.009 | 0.8416 |
| Mid-life**^†^** | 0.233 | **3.32E-07** |  | -0.21 | **4.02E-06** |  | 0.06 | 0.192 |  | -0.058 | 0.2075 |  | -0.062 | 0.129 |
| Late-life**^†^** | 0.303 | **3.11E-08** |  | -0.058 | 0.2958 |  | 0.056 | 0.3193 |  | -0.117 | **0.0343** |  | -0.072 | 0.153 |
| *APOE ε4* non-carriers**^‡^** | 0.295 | **6.45E-14** |  | -1.11 | **0.0056** |  | -0.011 | 0.7747 |  | -0.139 | **0.00049** |  | -0.043 | 0.207 |
| *APOE ε4* carriers**^‡^** | 0.167 | 0.0668 |  | -0.177 | **0.0398** |  | 0.159 | 0.0562 |  | -0.009 | 0.0855 |  | 0.04 | 0.5996 |

**Table S5: Association of CSF sTREM2 and amyloid-related biomarkers and cognitive function**

Independent variable was MDS. Dependent variables, including CSF amyloid markers and cognitive function, were normalized by log-transformed and standardized by z-scale. Significant effects (p<0.05) are shown in bold.

#Adjusting for age, sex, APOE ε4 status, and education.

*Adjusting for age, APOE ε4 status, and education.

†Adjusting for sex, APOE ε4 status, and education.

‡Adjusting for age, sex, and education.

Abbreviations: Aβ, amyloid-β; P-tau, phosphorylated tau; CSF, cerebrospinal fluid; APOE, apolipoprotein E; MDS, minimal depressive symptom; MMSE, Mini-Mental State examination.

**Figure legends**


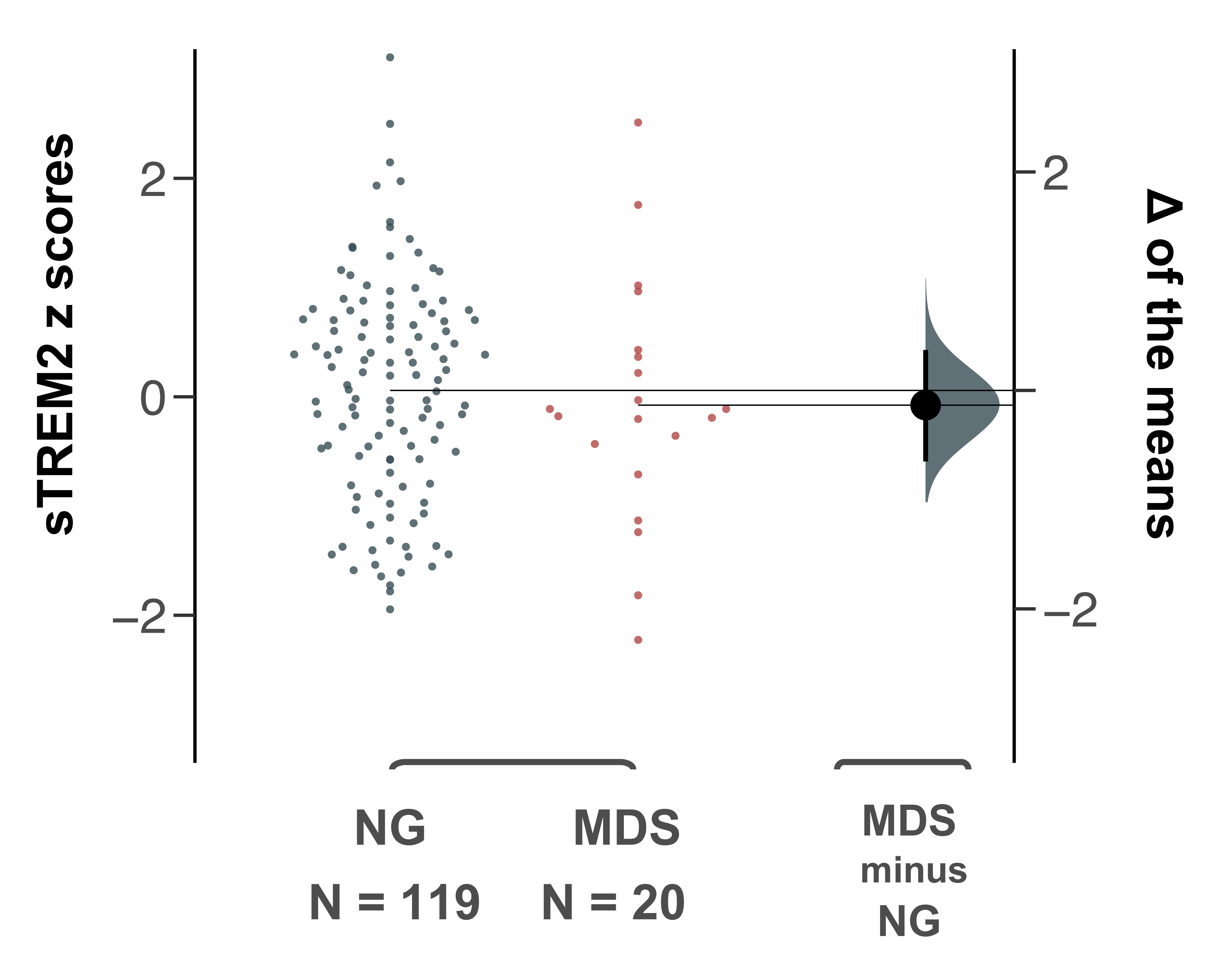


**Figure S1: Differences in concentration of CSF sTREM2 in APOE ε4 carriers**

CSF sTREM2 was not significant different in APOE ε4 carriers with or without MDSs. CSF, cerebrospinal fluid; sTREM2, soluble of trigging receptor expressed on myeloid cells 2; MDS, minimal depressive symptom; APOE, apolipoprotein E.


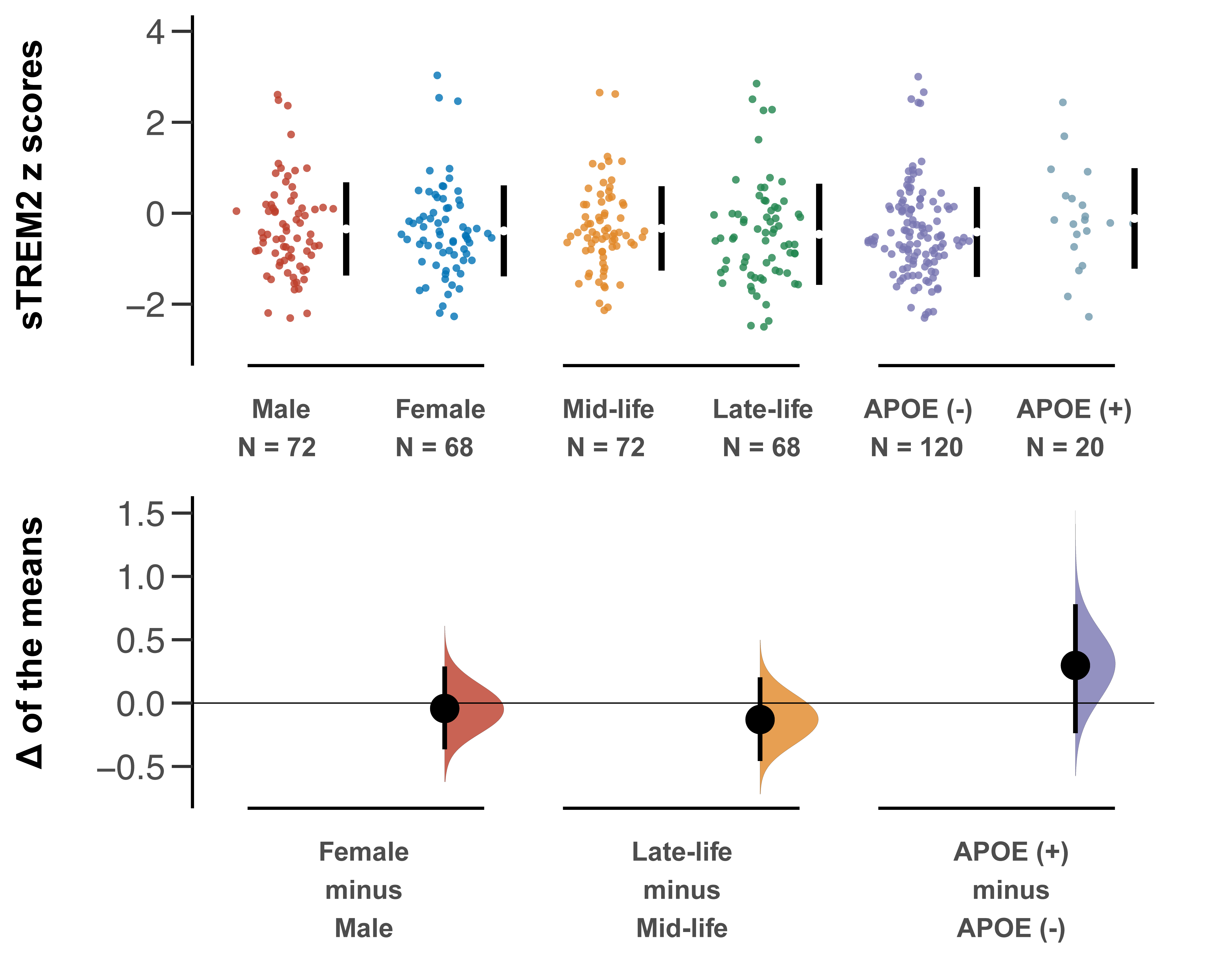


**Figure S2: Differences in concentration CSF sTREM2 concertation in subgroups with MDSs**

The total samples were categorized into six subgroups including male, female, mid-life, late-life, *APOE ε4* non-carrier, and *APOE ε4* carrier group. Levels of CSF sTREM2 concentration did not show any significant differences in MDS and normal participants in subgroups. The upper panel shows the distribution of raw data points for the entire dataset and the lower panel shows the differences by using 5,000 bootstrapped resamples and with difference-axis origin aligned to the mean of the normal group distribution. For each estimation plot: black dot represents mean difference (as indicated in lower panel); black ticks indicate 95% confidence interval; and shaded area represents bootstrapped sampling error distribution. CSF, cerebrospinal fluid; sTREM2, soluble of trigging receptor expressed on myeloid cells 2; MDS, minimal depressive symptom; APOE, apolipoprotein E.


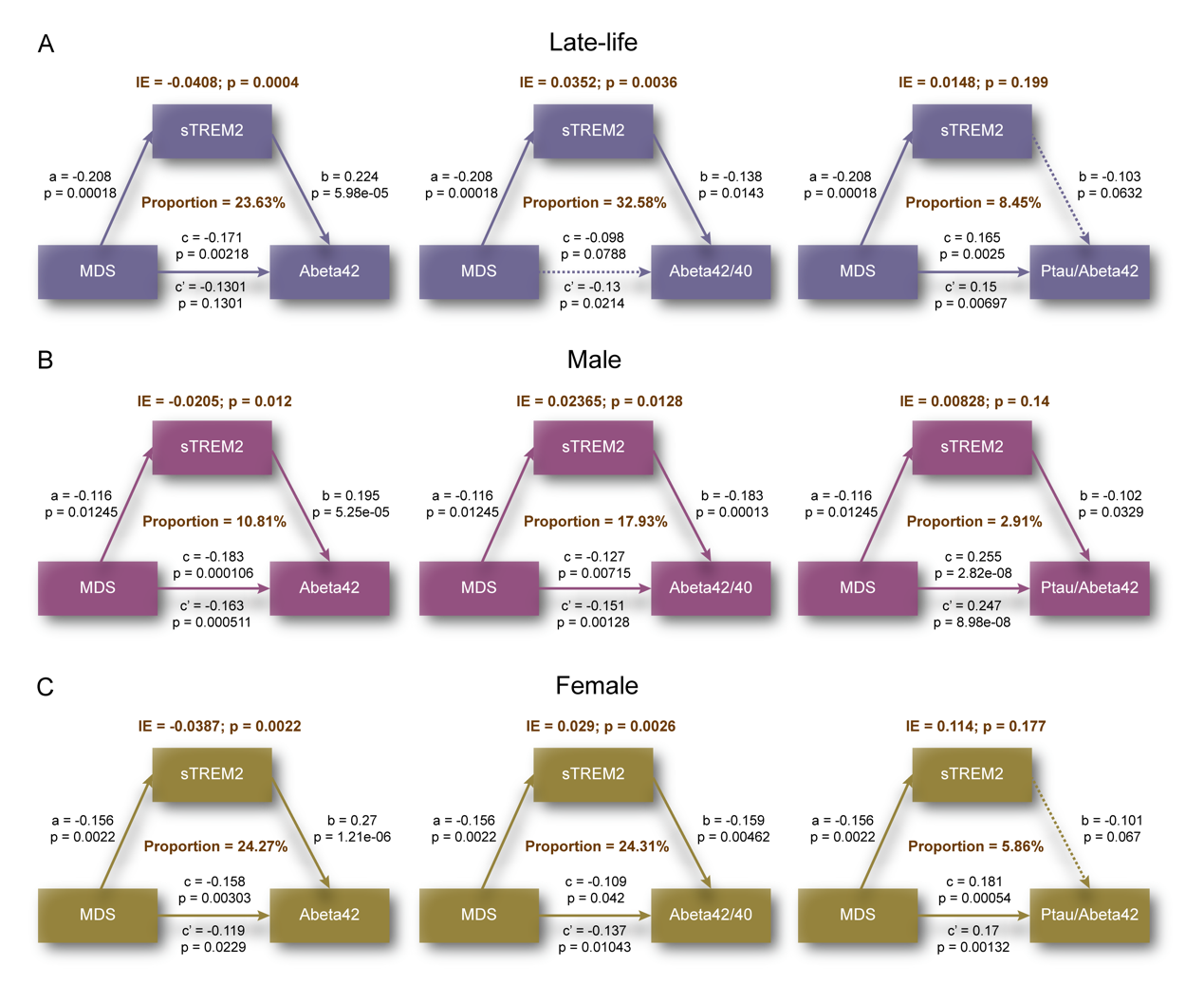


**Figure S3:** **Mediation analyses in late-life, male, and female**

Models of mediation for MDS, CSF sTREM2, and CSF amyloid markers (ie. Aβ42, Aβ42/40, Ptau/Aβ42), with MDS as independent variable and CSF sTREM2 as mediator and CSF amyloid markers as dependent variables. The mediation models were tested separately in late-life (A), male (B), and female (C). CSF, cerebrospinal fluid; sTREM2, soluble of trigging receptor expressed on myeloid cells 2; MDS, minimal depressive symptom; APOE, apolipoprotein E. Aβ, amyloid-β; P-tau, phosphorylated tau; IE, indirect effect.


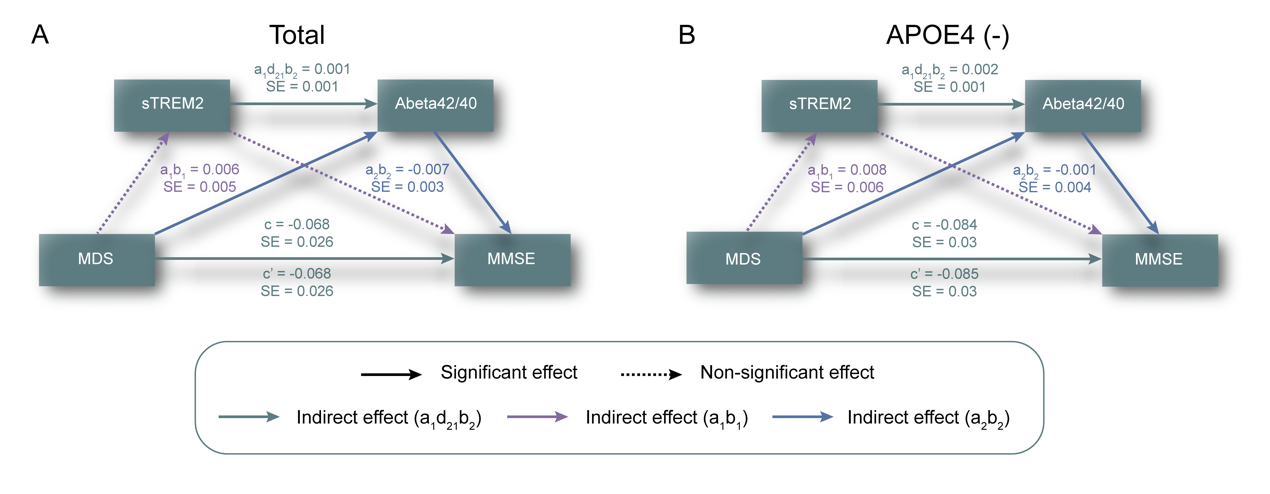


**Figure S4: Mediation analyses of sTREM2-amyloid pathways**

Models analyses were used to examine the mediation effects of CSF sTREM2 and CSF Aβ42/40 on MMSE as cognitive scores in total participants (A) and APOE ε4 non-carriers (B). Of which dotted line indicated non-significant indirect effect; solid line indicated significant indirect effect; and indirect effects are shown in purple (path a_1_b_1_, mediation effect of CSF sTREM2), in blue (path a_2_b_2_, mediation effect of Aβ42/40), and in green (path a_1_d_21_b_2_, mediation effect of CSF sTREM2 and Aβ42/40). CSF, cerebrospinal fluid; sTREM2, soluble of trigging receptor expressed on myeloid cells 2; MDS, minimal depressive symptom; APOE, apolipoprotein E. Aβ, amyloid-β; P-tau, phosphorylated tau; MMSE, Mini-Mental State examination; IE, indirect effect.
